# LncRNA CRNDE Promotes ATG4B-Mediated Autophagy and Alleviates the Sensitivity of Sorafenib in Hepatocellular Carcinoma Cells

**DOI:** 10.3389/fcell.2021.687524

**Published:** 2021-08-02

**Authors:** Lingxi Chen, Liangbo Sun, Xufang Dai, Tao Li, Xiaojing Yan, Yueting Zhang, Hanxi Xiao, Xiaodong Shen, Gang Huang, Wei Xiang, Yan Zhang, Dehong Tan, Shiming Yang, Yongzhan Nie, Xuequan Huang, Jiqin Lian, Fengtian He

**Affiliations:** ^1^Department of Biochemistry and Molecular Biology, College of Basic Medical Sciences, Army Medical University, Chongqing, China; ^2^Department of Clinical Biochemistry, Faculty of Pharmacy and Laboratory Medicine, Army Medical University, Chongqing, China; ^3^College of Educational Science, Chongqing Normal University, Chongqing, China; ^4^Institute of Hepatopancreatobiliary Surgery, Southwest Hospital, Army Medical University, Chongqing, China; ^5^Department of Gastroenterology, Xinqiao Hospital, Army Medical University, Chongqing, China; ^6^State Key Laboratory of Cancer Biology, Air Force Medical University, Xi’an, China; ^7^Center of Minimally Invasive Intervention, Southwest Hospital, Army Medical University, Chongqing, China

**Keywords:** hepatocellular carcinoma, CRNDE, ATG4B, autophagy, sorafenib

## Abstract

Autophagy is closely related to the growth and drug resistance of cancer cells, and autophagy related 4B (ATG4B) performs a crucial role in the process of autophagy. The long non-coding RNA (lncRNA) colorectal neoplasia differentially expressed (CRNDE) promotes the progression of hepatocellular carcinoma (HCC), but it is unclear whether the tumor-promoting effect of CRNDE is associated with the regulation of ATG4B and autophagy. Herein, we for the first time demonstrated that CRNDE triggered autophagy via upregulating ATG4B in HCC cells. Mechanistically, CRNDE enhanced the stability of ATG4B mRNA by sequestrating miR-543, leading to the elevation of ATG4B and autophagy in HCC cells. Moreover, sorafenib induced CRNDE and ATG4B as well as autophagy in HCC cells. Knockdown of CRNDE sensitized HCC cells to sorafenib *in vitro* and *in vivo*. Collectively, these results reveal that CRNDE drives ATG4B-mediated autophagy, which attenuates the sensitivity of sorafenib in HCC cells, suggesting that the pathway CRNDE/ATG4B/autophagy may be a novel target to develop sensitizing measures of sorafenib in HCC treatment.

## Introduction

Hepatocellular carcinoma (HCC) is one of the most common malignant tumors in human digestive system with high incidence and low survival rate (Siegel et al., 2020; Sung et al., 2021). The main strategies for HCC treatment with proven survival benefit include surgical resection, ablation, transplantation, chemotherapy, targeted therapy, and so on (Forner et al., 2018). In recent years, therapeutic reagents are increasingly used to treat HCC, but the efficacy tends to be limited due to acquired drug resistance (Llovet et al., 2018; Yang et al., 2019). Autophagy, a lysosome-mediated conserved process which degrades and recycles cellular proteins and organelles in response to starvation or cellular stresses, plays an essential part in maintaining intracellular homeostasis. Mounting studies have verified that autophagy attenuates the sensitivity of therapeutic drugs such as sorafenib, 5-fluorouracil (5-FU) and oxaliplatin, ultimately protecting cancer cells from death (Guo et al., 2014; Xiong et al., 2017; Li W. et al., 2019). Therefore, it is of great significance to understand the specific regulating mechanisms of autophagy in drug resistance and interfere with protective autophagy in order to improve the drug sensitivity in HCC.

Autophagy related 4B (ATG4B), a member of the autophagin protein family, performs a critical role in regulating autophagy in mammalian cells. In the process of autophagosome formation, C-terminal arginine residue of the cytoplasmic light chain 3 (LC3) is proteolytically cleaved to generate LC3-I, which subsequently conjugates with phosphatidylethanolamine and converts to membrane-bound LC3-II (Mizushima, 2007). The cysteine protease ATG4 is essential for activation of LC3 precursor and delipidation of LC3-II (Satoo et al., 2009). Among the four isoforms of ATG4 (4A, 4B, 4C, and 4D), ATG4B exhibits the dominant proteolysis activity in autophagic homeostasis (Mizushima, 2020). Recent studies have shown that ATG4B promotes the proliferation and progression of various tumor cells, and enhances the drug resistance of HCC cells through inducing protective autophagy (Rothe et al., 2014; Zhang et al., 2017; Agrotis and Ketteler, 2019; Liu P. F. et al., 2019). However, it is not well known about the molecular mechanisms underlying the regulation of ATG4B in HCC cells.

Long non-coding RNAs (lncRNAs), a subgroup of non-coding transcripts longer than 200 nucleotides in length, perform functions of regulating gene expression directly or indirectly at several levels such as transcriptional, post transcriptional, translational, and epigenetic regulation (Ulitsky and Bartel, 2013). An increasing number of lncRNAs have been reported to play a vital role in the carcinogenesis and development of HCC (Batista and Chang, 2013; Huang et al., 2020). As a new member of lncRNA family, CRNDE (colorectal neoplasia differentially expressed) is initially identified as the specifically upregulated lncRNA in human colorectal cancer, and afterward confirmed to be highly expressed in many other malignancies including HCC (Ellis et al., 2012; Wang et al., 2015; Xie et al., 2018). CRNDE promotes the growth and invasion of HCC cells by regulating multiple signaling pathways, such as PI3K/Akt, BCAT1, MAPK, and Wnt/β-catenin pathways (Tang et al., 2018; Wang et al., 2018; Zhu et al., 2018; Ji et al., 2019), suggesting that CRNDE may act as an oncogenic lncRNA which could be served as a potential target for HCC therapy (Zhang et al., 2018). Nevertheless, whether CRNDE is associated with the regulation of ATG4B and autophagy in HCC cells, and the roles of CRNDE in response to therapeutic agents remain unclear.

In the present study, we for the first time revealed that CRNDE caused autophagy in HCC cells via elevating ATG4B. Mechanistic research showed that CRNDE increased the stability of ATG4B mRNA through sequestrating miR-543, leading to the upregulation of ATG4B and the enhancement of autophagy in HCC cells. Furthermore, sorafenib induced CRNDE and ATG4B as well as autophagy, while inhibiting CRNDE/ATG4B/autophagy pathway sensitized HCC cells to sorafenib. The *in vivo* experiments in nude mice showed that knockdown of CRNDE strengthened the anti-HCC effect of sorafenib. Taken together, these findings demonstrate that CRNDE promotes ATG4B-mediated autophagy, which alleviates the sensitivity of sorafenib in HCC cells, suggesting that the pathway CRNDE/ATG4B/autophagy may be a promising target to improve the sensitivity of sorafenib against HCC.

## Materials and Methods

### Clinical Specimens

Human HCC specimens and the adjacent non-cancerous tissues were collected from Southwest Hospital of Army Medical University with informed consent. The study was approved by the Ethics Committee of Army Medical University.

### The Cancer Genome Atlas Data

Hepatocellular carcinoma RNA-Seq data and the related clinical data were from TCGA database^1^.

### Cell Culture

Human HCC cell lines PLC, HepG2, and Hep3B were purchased from the American Type Culture Collection (Manassas, VA, United States). Huh7 and SMMC7721 cell lines, and the relatively normal hepatic cell line THLE-3 were from the Cell Bank of Chinese Academy of Sciences (Shanghai, China). The cells were cultured in high-glucose DMEM (Gibco, Carlsbad, CA, United States) containing 10% fetal bovine serum (FBS) at 37°C in a humid incubator with 5% CO_2_.

### Quantitative Real-Time PCR

Total RNA from HCC cells or tissues was extracted using total RNA extraction kit (BioFlux, Hangzhou, China) according to the manufacturer’s instructions. For nuclear/cytoplasmic separation assay, cytoplasmic and nuclear RNA were separately isolated using PARIS kit (Invitrogen, Carlsbad, CA, United States) according to the manufacturer’s protocol. The RNA was reversely transcribed to first-strand cDNA using PrimeScript RT reagent kit (Takara, Dalian, China). qPCR was performed with SYBR qPCR master mix (Takara), taking GAPDH as the internal reference. Reverse transcription and qPCR of miRNAs were conducted with All-in-One miRNA qRT-PCR detection kit (GeneCopoeia, Guangzhou, China), taking U6 RNA as the internal control. The primers were listed in Supplementary Table 1.

### Western Blot

Total proteins from HCC cells or tissues were extracted with RIPA lysis buffer (Beyotime, Shanghai, China) and the protein concentrations were detected using BCA protein assay kit (Beyotime). Then Western blot was performed as previously described (Xiong et al., 2017). The primary antibodies anti-ATG4B and anti-GAPDH were from Proteintech (Chicago, IL, United States), anti-PARP and anti-SQSTM1/p62 were from CST (Beverly, MA, United States), and anti-LC3 was from Sigma-Aldrich (St Louis, MO, United States).

### Construction of Plasmids

The DNA fragments encoding the wild type and the mutant human CRNDE were chemically synthesized by Sangon Biotech (Shanghai, China), and separately inserted into pcDNA3.1(+) (pcDNA3.1) expression vector (Invitrogen) after digestion with *Eco*RI (Takara) and *Bam*HI (Takara). The recombinant plasmids were named as pcDNA-CRNDE and pcDNA-CRNDE-mut, respectively. pcDNA-CRNDE-mut contained mutations of the predicted miR-543 binding site in CRNDE sequence (1008 CTTTATTGGATTGAATGAATGTTT 1031, the underlined nucleotides were mutated). The DNA fragments encoding the wild type and the mutant 3′-UTR of human ATG4B mRNA were separately synthesized by Sangon Biotech, digested with *Sac*I (Takara) and *Sal*I (Takara), and then cloned into pmir-GLO reporter vector (Thermo Scientific, Waltham, MA, United States). The reconstructed plasmids were named as pmir-ATG4B and pmir-ATG4B-mut, respectively. pmir-ATG4B-mut contained mutations of the putative miR-543 binding site in 3′-UTR of ATG4B mRNA (1565 TGTCAGACACAGACATGAATTTCT 1588, the underlined nucleotides were mutated). The overexpression plasmid pCMV-ATG4B was bought from Lab Cell Biotechnology (Chongqing, China).

### Transfection Assay

The siRNAs separately targeting human CRNDE and ATG4B, and the control siRNA were synthesized by Lab Cell Biotechnology. Mimics and inhibitor of miRNAs, and the corresponding negative controls were purchased from GeneCopoeia. The cells were transfected using Lipofectamine 3000 (Invitrogen) according to the manufacturer’s instructions. The sequences of siRNA were presented in Supplementary Table 2, and the sequences of miRNA mimics and inhibitor were listed in Supplementary Table 3.

### Transmission Electron Microscopy

The cells were seeded into the dishes (6 cm in diameter) and transfected with pcDNA-CRNDE (or pcDNA3.1) using Lipofectamine 3000 (Invitrogen) for 24 h. Then the cells were collected, fixed with 2.5% glutaraldehyde for 4 h and 1% osmium acid for 2 h. After gradient dehydration and penetration, the samples were embedded in Epon-Araldite resin, followed by high-temperature polymerization. Subsequently, the samples were cut into ultrathin sections, counterstained with uranium acetate and lead citrate, and observed under a transmission electron microscope (JEOL, Japan).

### GFP-LC3 Analysis

HCC cells were seeded into 12-well plates, co-transfected with GFP-LC3 plasmid and pcDNA-CRNDE (or pcDNA3.1) or ATG4B siRNA (or control siRNA) with Lipofectamine 3000 (Invitrogen) for 24 h, and then fixed with 4% formaldehyde for 10 min. Next, the cells were observed for five views randomly, and the number of positive autophagic cells containing five or more GFP-LC3 puncta from 50 cells for each view was counted under a fluorescence microscope (OlympusIX81, Tokyo, Japan). The representative cell images were photographed using a laser confocal microscope (Carl Zeiss AG, Oberkochen, Germany). GFP-LC3 plasmid was kindly offered by Dr. N. Mizushima and Dr. T. Yoshimori (Osaka University, Japan).

### Fluorescent Assay of Autophagosomes

Autophagy in live cells was analyzed using Cyto-ID autophagy detection kit (Enzo, NY, United States) with proprietary probes specifically staining autophagosomes according to the manufacturer’s protocol. Briefly, the cells were harvested, resuspended in 500 μL freshly diluted Cyto-ID green detection reagent and incubated at room temperature for 30 min in dark. Then fluorescence intensity was analyzed using a flow cytometer (Beckman, CA, United States).

### RNA Fluorescence *in situ* Hybridization

The Cy3-labeled probes (red fluorescent signal) for RNA FISH of CRNDE, 18S RNA and U6 RNA were synthesized by RiboBio (Guangzhou, China). RNA FISH was performed using fluorescent *in situ* hybridization kit (RiboBio) according to the manufacturer’s instructions. DAPI was used for counterstaining of nuclei (blue fluorescent signal). The cells were photographed under a fluorescence microscope (OlympusIX81).

### RNA Immunoprecipitation Assay

RNA Immunoprecipitation (RIP) assay was performed using EZ-Magna RIP kit (Millipore/Merck, Darmstadt, Germany) according to the manufacturer’s protocol. Briefly, the cells were lysed and incubated with antibody-coated beads at 4°C overnight. Subsequently, the co-immunoprecipitates were treated with proteinase K at 55°C for 30 min. RNA was purified with phenol:chloroform:isoamyl alcohol (125:24:1), precipitated with ethanol overnight, and reversely transcribed into cDNA using PrimeScript RT reagent kit (Takara). Then the cDNA was analyzed by qPCR with SYBR qPCR master mix (Takara).

### Dual-Luciferase Reporter Assay

HCC cells were seeded into 24-well plates and co-transfected with the indicated luciferase reporter plasmids and miRNA mimics, siRNA, expression plasmid, or the corresponding control using Lipofectamine 3000 (Invitrogen) for 24 h. Then the cells were lysed, and the reporter assay was performed using dual-luciferase reporter assay kit (GeneCopoeia) according to the manufacturer’s instructions.

### Cell Viability Assay

The cell viability assay was performed using cell counting kit-8 (CCK-8; Beyotime) according to the manufacture’s protocol. Briefly, HCC cells were seeded into 48-well plates, followed by different treatments. Then 20 μL CCK-8 reagent was added to each well and incubated at 37°C for 1 h. Subsequently, the OD values at 450 nm were examined using a microplate reader (Molecular Devices, Sunnyvale, CA, United States).

### Cell Apoptosis Detection

Cell apoptosis was detected using the two methods: (1) The cells were fixed with 4% paraformaldehyde for 10 min and stained with Hoechst 33258 (Beyotime) for 10 min in dark. Then the cells were photographed under a fluorescence microscope (OlympusIX81) and the apoptotic cells were characterized by the nuclear morphology changes; (2) The cells were harvested and stained with Annexin-V/FITC and PI (BD, San Diego, CA, United States) at room temperature for 15 min in dark. Then the apoptotic cells were analyzed using a flow cytometer (Beckman).

### Transwell Migration Assay

The cells were suspended with DMEM containing 1% FBS (1 × 10^6^ cells/mL), and then seeded into transwell chambers (Corning, NY, United States) in 24-well plates with DMEM containing 10% FBS. After incubation at 37°C for 24 h, the cells were fixed with 70% methanol and stained with 0.1% crystal violet. Next, the migrated cells were observed and counted for five random views under a fluorescence microscope (OlympusIX81).

### Animal Experiments

Male nude mice (6 weeks old) were obtained from Beijing Huafukang Bioscience (Beijing, China). Lentivirus system was used to construct the HepG2 cell lines with (or without) stable knockdown of CRNDE (LV-shCRNDE or LV-NC) as previously described (Xiong et al., 2017). 5 × 10^6^ cells in 0.1 mL PBS (for each mouse) were subcutaneously injected into the right flank of each mouse (*n* = 10 per group). After a week, the mice in each group were randomized into two subgroups (*n* = 5) and given daily administration of sorafenib (Selleckchem, Houston, TX, United States; 30 mg/kg) or vehicle control by gavage. The size of xenograft tumors was measured every 3 days and the volume was calculated as width^2^ × length × 1/2. Twenty-five days after inoculation, the mice were sacrificed and the tumors were excised for photographing and weighting. The cell apoptosis in the xenograft tumors was determined using TUNEL staining, and the levels of corresponding RNAs and proteins were analyzed by qPCR, immunohistochemical (IHC) staining and Western blot, respectively. All procedures in the animal experiments were performed under the guidelines of Laboratory Animal Center of Army Medical University and approved by the Ethics Committee of Army Medical University (No. 20190078).

### IHC Staining and TUNEL Assay

The xenograft tumor tissues were fixed with 4% paraformaldehyde, and then embedded in paraffin and sectioned. Next, the tumor sections were immunostained using histostain-plus kit (Zhongshan Biotechnologies, Beijing, China) according to the manufacturer’s instructions, counterstained with hematoxylin and photographed under a microscope (OlympusIX81). For apoptosis analysis of the xenograft tumors, TUNEL assay was conducted according to the manufacturer’s protocol (Zhongshan Biotechnologies) and the tumor sections were visualized using a laser confocal microscope (Carl Zeiss AG).

### Statistical Analysis

The data were presented as means ± SD. Student’s *t*-test was used for the comparisons between two independent groups. Pearson’s test was employed for the analysis of the correlation between CRNDE and ATG4B levels. *P* < 0.05 was considered statistically significant.

## Results

### CRNDE Upregulates ATG4B in HCC Cells

**FIGURE 1:**
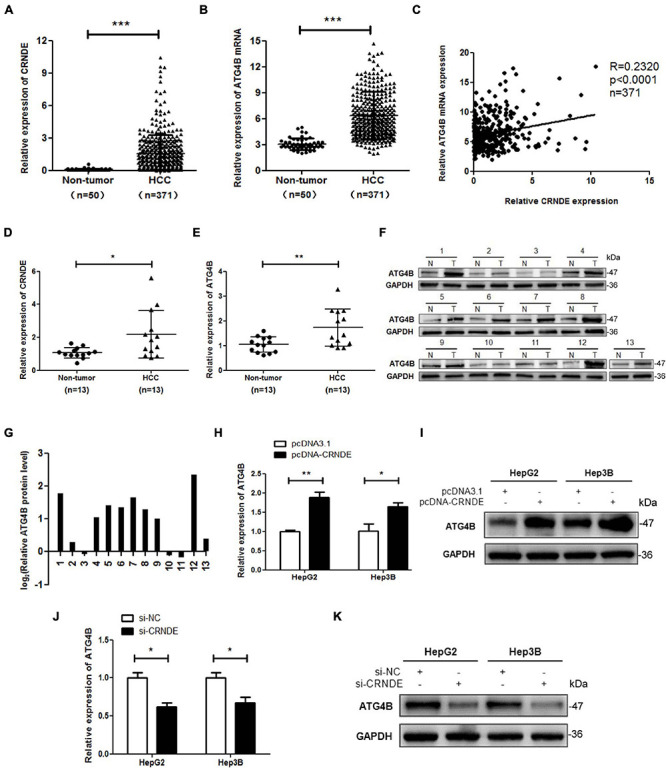
CRNDE elevates ATG4B in HCC cells. **(A,B)** Expression analysis of CRNDE and ATG4B in HCC tissues and non-cancerous liver tissues using TCGA database. **(C)** Analysis of the correlation between CRNDE and ATG4B mRNA levels in HCC tissues using TCGA database. **(D–F)** The levels of CRNDE, ATG4B mRNA and protein in 13 pairs of HCC tissues (T) and the adjacent non-cancerous liver tissues (N) were separately assayed by qPCR **(D,E)** and Western blot **(F)**. **(G)** The protein band density in F was analyzed using Image Lab software and the relative ATG4B protein level (ATG4B/GAPDH ratio) was transformed to Log_2_ (T/N). **(H–K)** HepG2 and Hep3B cells were transfected with pcDNA-CRNDE (or pcDNA3.1) **(H,I)** or si-CRNDE (or si-NC) **(J,K)** for 24 h, then the levels of ATG4B mRNA and protein were separately detected by qPCR **(H,J)** and Western blot **(I,K)**. pcDNA-CRNDE: CRNDE expression vector; pcDNA3.1: control vector pcDNA3.1(+); si-CRNDE: the siRNA for CRNDE; si-NC: control siRNA; *P < 0.05; **P < 0.01; ***P < 0.001.

To preliminarily explore the relationship between CRNDE and ATG4B, TCGA database was used to analyze the levels of CRNDE and ATG4B in HCC tissues. The results showed that both CRNDE and ATG4B mRNA levels in HCC tissues were significantly higher than those in non-cancerous liver tissues (Figures 1A,B). The correlation analysis revealed that the level of CRNDE was positively correlated with that of ATG4B in HCC tissues (Figure 1C). Furthermore, high levels of CRNDE and ATG4B mRNA were associated with a shorter overall survival of HCC patients (Supplementary Figure 1). The results of qPCR (Figures 1D,E) and Western blot (Figures 1F,G) showed that the levels of CRNDE as well as ATG4B mRNA and protein in HCC tissues were higher than those in the corresponding adjacent non-cancerous liver tissues. In addition, the levels of CRNDE and ATG4B in 5 HCC cell lines (SMMC-7721, HepG2, Hep3B, Huh7, and PLC) were also higher than those in relatively normal hepatic cell line THLE-3 (Supplementary Figure 2). Subsequently, we investigated whether CRNDE could regulate ATG4B in HCC cells using CRNDE overexpression and silencing strategies (Supplementary Figure 3). As shown in Figures 1H,I, overexpression of CRNDE markedly increased ATG4B at both mRNA and protein levels, whereas silence of CRNDE dramatically reduced the mRNA and protein levels of ATG4B in HCC cells (Figures 1J,K). Taken together, these results indicate that CRNDE elevates ATG4B in HCC cells.

### CRNDE Enhances Autophagy via Upregulating ATG4B

**FIGURE 2:**
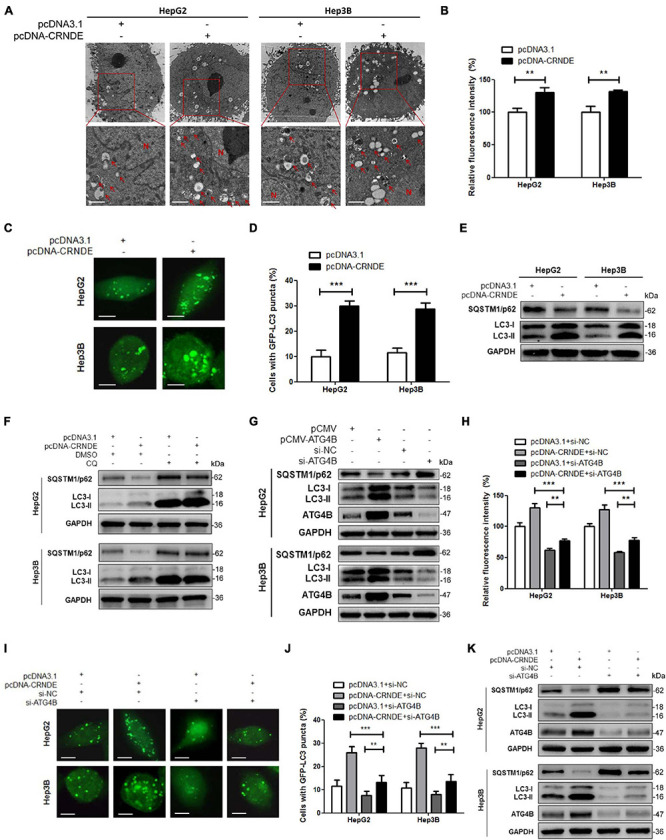
CRNDE induces autophagy in HCC cells through upregulating ATG4B. **(A,B)** HepG2 and Hep3B cells were transfected with pcDNA-CRNDE (or pcDNA3.1) for 24 h, then autophagic vesicles in the cells were observed by TEM (scale bar: 1 μm; N, nuclei; red arrows indicate the typical autophagosomes or autolysosomes) **(A)**, and fluorescence intensity of autophagosomes was analyzed by flow cytometry **(B)**. **(C,D)** HepG2 and Hep3B cells were co-transfected with pcDNA-CRNDE (or pcDNA3.1) and GFP-LC3 vector for 24 h, then the green fluorescent GFP-LC3 puncta in the cells were observed under a laser confocal microscope (scale bar: 5 μm) **(C)**, and the percentage of the cells containing five or more GFP-LC3 puncta was quantified using a fluorescence microscope **(D)**. **(E)** HepG2 and Hep3B cells were transfected as in A, and then the levels of SQSTM1/p62 and LC3 were measured by Western blot. **(F)** HepG2 and Hep3B cells were transfected with pcDNA-CRNDE (or pcDNA3.1) in the presence of 20 μM chloroquine (CQ) (or vehicle control DMSO) for 24 h, then the levels of SQSTM1/p62 and LC3 were detected by Western blot. **(G)** HepG2 and Hep3B cells were transfected with pCMV-ATG4B (or pCMV) or si-ATG4B (or si-NC) for 24 h, then the levels of SQSTM1/p62, LC3, and ATG4B were examined by Western blot. **(H)** HepG2 and Hep3B cells were co-transfected with pcDNA-CRNDE (or pcDNA3.1) and si-ATG4B (or si-NC) for 24 h, then fluorescence intensity of autophagosomes was analyzed by flow cytometry. **(I,J)** HepG2 and Hep3B cells were co-transfected with pcDNA-CRNDE (or pcDNA3.1) and si-ATG4B (or si-NC) in the presence of GFP-LC3 vector for 24 h, then the green fluorescent GFP-LC3 puncta in the cells were observed under a laser confocal microscope (scale bar: 5 μm) **(I)**, and the percentage of the cells containing five or more GFP-LC3 puncta was quantified using a fluorescence microscope **(J)**. **(K)** HepG2 and Hep3B cells were co-transfected as in H, and then the levels of SQSTM1/p62, LC3, and ATG4B were determined by Western blot. pCMV-ATG4B: ATG4B expression vector; pCMV: pCMV vector (control vector); si-ATG4B: the siRNA for ATG4B; pcDNA-CRNDE, pcDNA3.1 and si-NC were the same as the description in Figure 1; **P < 0.01; ***P < 0.001.

As shown in Figures 2A–E and Supplementary Figure 4, overexpression of CRNDE significantly increased the formation of autophagic vesicles (Figure 2A), fluorescence intensity of autophagosome (Figure 2B), GFP-LC3 puncta (Figures 2C,D and Supplementary Figure 4) and LC3-II accumulation, simultaneously decreased SQSTM1/p62 (Figure 2E). Moreover, autophagy inhibitor chloroquine (CQ) dramatically enhanced CRNDE-induced LC3-II accumulation while reversed CRNDE-mediated SQSTM1/p62 reduction (Figure 2F and Supplementary Figure 5). These results indicate that CRNDE triggers autophagy in HCC cells. Subsequently, the effect of ATG4B on autophagy in HCC cells was investigated using ATG4B overexpression and silencing methods (Supplementary Figure 6A). As shown in Figure 2G, overexpression of ATG4B increased LC3-II while decreased SQSTM1/p62, whereas knockdown of ATG4B reduced LC3-II while elevated SQSTM1/p62. Furthermore, silence of ATG4B remarkably ameliorated the CRNDE-increased fluorescence intensity of autophagosome (Figure 2H), GFP-LC3 puncta (Figures 2I,J and Supplementary Figure 6B) and LC3-II accumulation, while abolished the CRNDE-mediated decline of SQSTM1/p62 (Figure 2K and Supplementary Figure 6C). In brief, the above data confirm that CRNDE drives autophagy through upregulating ATG4B in HCC cells.

### CRNDE Promotes the Stability of ATG4B mRNA

**FIGURE 3:**
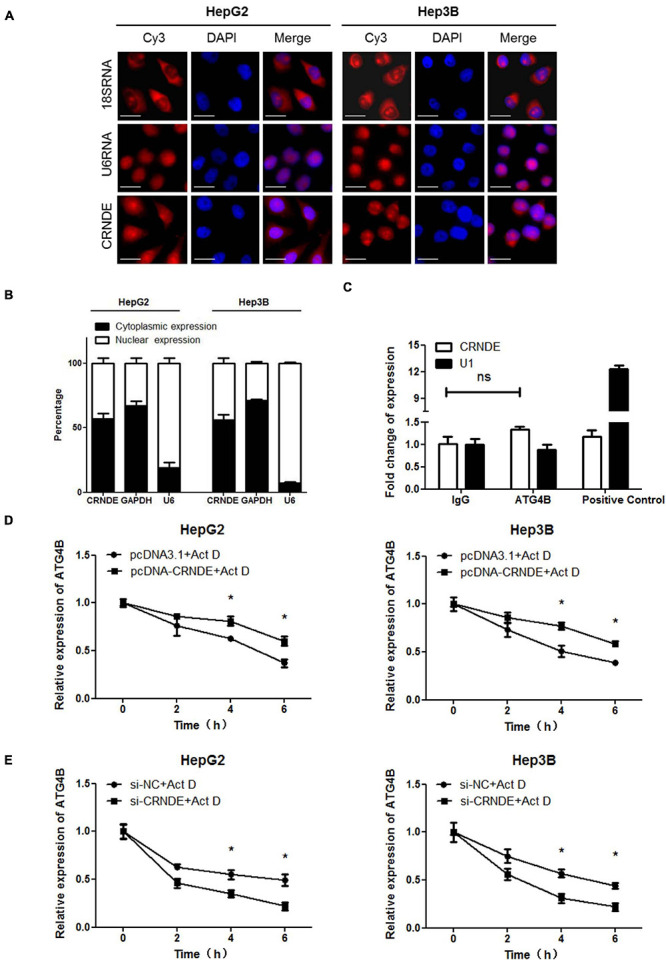
CRNDE enhances the stability of ATG4B mRNA. **(A)** Subcellular distribution of CRNDE in HCC cells was assayed by FISH (Scale bar: 10 μm). **(B)** The levels of nuclear and cytoplasmic CRNDE in HCC cells were tested by qPCR. GAPDH RNA and U6 RNA were used as cytoplasmic and nuclear RNA controls, respectively. **(C)** The level of CRNDE in the co-immunoprecipitates with anti-ATG4B antibody in HepG2 cells was examined by RIP assay. SNRNP70 (binding to U1 snRNA) and IgG were used as positive control and negative control, respectively. **(D,E)** After transfected with pcDNA-CRNDE (or pcDNA3.1) **(D)** or si-CRNDE (or si-NC) **(E)** for 18 h, HepG2 and Hep3B cells were treated with 5 μg/mL actinomycin D (Act D) for the indicated times, and then the level of ATG4B mRNA was detected by qPCR. pcDNA-CRNDE, pcDNA3.1, si-CRNDE and si-NC were the same as the description in Figure 1; ns, no significance; *P < 0.05.

First, the distribution of CRNDE was analyzed. As shown in Figure 3A, RNA FISH showed that CRNDE located in both cytoplasm and nucleus of HCC cells. Nuclear/cytoplasmic separation assay further indicated that the majority of CRNDE distributed in cytoplasm and the rest located in nucleus (Figure 3B). Second, it has been reported that lncRNAs could directly bind to the corresponding proteins to exert their functions (Tsagakis et al., 2020), thus RIP assay was performed to explore whether CRNDE could directly bind to ATG4B protein. However, the result showed that CRNDE was not specifically enriched in the RNAs co-immunoprecipitated with ATG4B (Figure 3C). Third, the influence of CRNDE on ATG4B mRNA stability in HCC cells were examined. As shown in Figure 3D and Supplementary Figure 7A, in the presence of the transcription inhibitor actinomycin D (Act D), the level of ATG4B mRNA in CRNDE-overexpressed cells was significantly higher than that in the control cells. Conversely, the level of ATG4B mRNA in CRNDE-knockdown cells was markedly lower than that in the corresponding control cells in the presence of Act D (Figure 3E and Supplementary Figure 7B). These findings reveal that CRNDE upregulates ATG4B through increasing its mRNA stability in HCC cells.

### miR-543 Downregulates ATG4B by Targeting Its 3′-UTR

**FIGURE 4:**
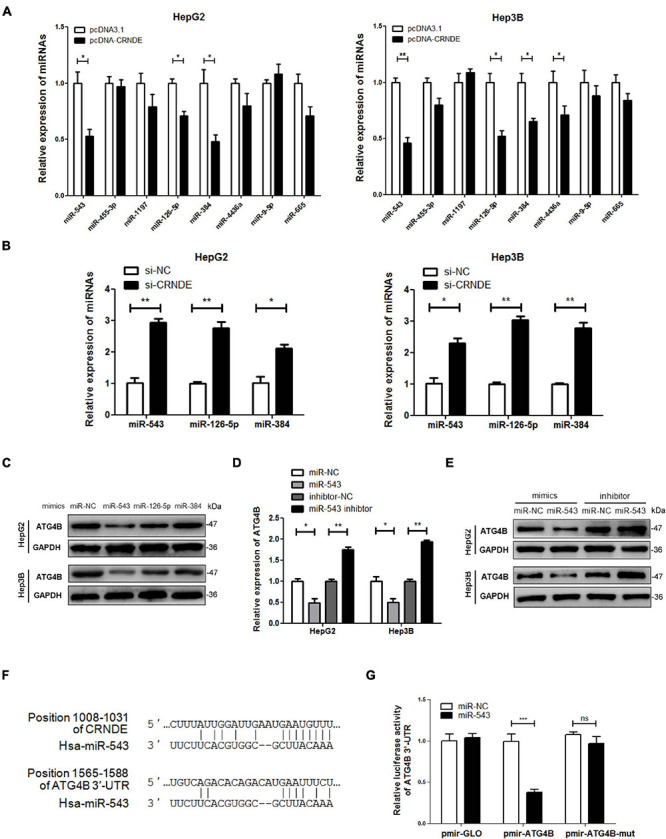
miR-543 downregulates ATG4B by targeting its 3′-UTR. **(A,B)** HepG2 and Hep3B cells were transfected with pcDNA-CRNDE (or pcDNA3.1) **(A)** or si-CRNDE (or si-NC) **(B)** for 24 h, then the indicated miRNAs were analyzed by qPCR. **(C)** HepG2 and Hep3B cells were separately transfected with the mimics of the three candidate miRNAs (or miR-NC) for 24 h, then ATG4B protein level was measured by Western blot. **(D,E)** HepG2 and Hep3B cells were transfected with miR-543 mimics (or miR-NC) or inhibitor (or inhibitor-NC) for 24 h, then the levels of ATG4B mRNA and protein were separately detected by qPCR (D) and Western blot **(E)**. **(F)** The predicted binding sites of miR-543 in CRNDE and 3′-UTR of ATG4B mRNA. **(G)** HepG2 cells were co-transfected with the luciferase reporter plasmid pmir-ATG4B (or pmir-ATG4B-mut, or pmirGLO) and miR-543 mimics (or miR-NC) for 24 h. Then the luciferase activities were determined by dual-luciferase reporter assay. The activity of firefly luciferase was normalized against that of rennilla luciferase, and the result was shown as relative luciferase activity. miR-NC: control mimics; inhibitor-NC: control inhibitor; pmir-ATG4B: reporter vector of 3′-UTR of ATG4B mRNA; pmir-ATG4B-mut: reporter vector of mutant 3′-UTR of ATG4B mRNA; pmir-GLO: pmir-GLO vector; pcDNA-CRNDE, pcDNA3.1, si-CRNDE and si-NC were the same as the description in Figure 1; ns, no significance; *P < 0.05; **P < 0.01; ***P < 0.001.

Previous research has shown that cytoplasmic lncRNA can function as a competing endogenous RNA (ceRNA) by sequestrating miRNA, thereby represses the expression of the miRNA-targeted mRNA (Cesana et al., 2011; Wang et al., 2013). Thus, the miRNAs potentially binding to both CRNDE and 3′-UTR of ATG4B mRNA were predicted using online bioinformatic tools StarBase^2^ and TargetScan^3^. Eight candidate miRNAs were picked out from the putative miRNAs and identified whether CRNDE was involved in their regulation. As shown in Figure 4A, overexpression of CRNDE decreased the levels of miR-543, miR-126-5p and miR-384 in HepG2 and Hep3B cells. In contrast, knockdown of CRNDE increased the levels of these three miRNAs (Figure 4B). However, only miR-543 mimics could dramatically reduce ATG4B protein (Figure 4C), while miR-543 inhibitor elevated ATG4B at both mRNA and protein levels (Figures 4D,E). Bioinformatic analysis showed that miR-543 might bind to CRNDE and target 3′-UTR of ATG4B mRNA, and the potential binding sites were shown in Figure 4F. Furthermore, luciferase reporter assay revealed that miR-543 mimics obviously suppressed the luciferase activity of pmir-ATG4B, but not pmir-ATG4B-mut (in which the binding site of miR-543 in 3′-UTR of ATG4B mRNA was mutated) (Figure 4G). Collectively, these results indicate that miR-543 directly targets 3′-UTR of ATG4B mRNA, leading to the downregulation of ATG4B.

### CRNDE Elevates ATG4B and Autophagy via Sequestrating miR-543

**FIGURE 5:**
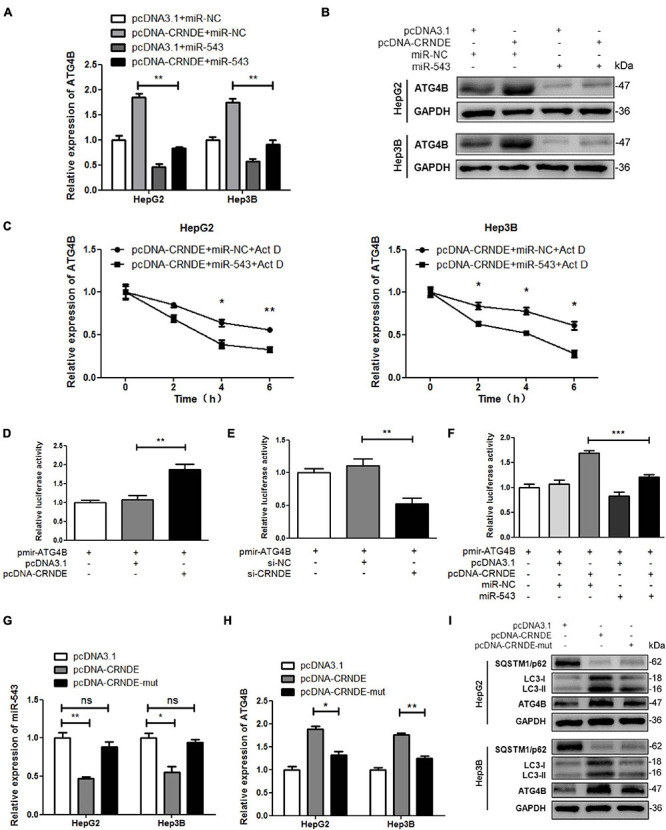
CRNDE upregulates ATG4B and promotes autophagy through sequestrating miR-543. **(A,B)** HepG2 and Hep3B cells were co-transfected with pcDNA-CRNDE (or pcDNA3.1) and miR-543 mimics (or miR-NC) for 24 h, then the levels of ATG4B mRNA and protein were separately determined by qPCR **(A)** and Western blot **(B)**. **(C)** After co-transfected with pcDNA-CRNDE (or pcDNA3.1) and miR-543 mimics (or miR-NC) for 18 h, HepG2 and Hep3B cells were treated with 5 μg/mL actinomycin D (Act D) for the indicated times, and then the level of ATG4B mRNA was quantified by qPCR. **(D,E)** HepG2 cells were transfected with pcDNA-CRNDE (or pcDNA3.1) **(D)** or si-CRNDE (or si-NC) **(E)** in the presence of pmir-ATG4B for 24 h. Then the luciferase activities were measured as described in Figure 4G. **(F)** HepG2 cells were co-transfected with pcDNA-CRNDE (or pcDNA3.1) and miR-543 mimics (or miR-NC) in the presence of pmir-ATG4B for 24 h. Then the luciferase activities were analyzed as described in Figure 4G. **(G–I)** HepG2 and Hep3B cells were separately transfected with pcDNA-CRNDE, pcDNA-CRNDE-mut, or pcDNA3.1 for 24 h. Subsequently, the levels of miR-543 and ATG4B mRNA were detected by qPCR **(G,H)**, and the level of ATG4B protein was examined by Western blot **(I)**. pcDNA-CRNDE-mut: the mutant CRNDE expression vector; pcDNA-CRNDE, pcDNA3.1, pmir-ATG4B, miR-NC, si-CRNDE, and si-NC were the same as the description in Figure 4; ns: no significance; *P < 0.05; **P < 0.01; ***P < 0.001.

As shown in Figures 5A–C, miR-543 remarkably alleviated the CRNDE-triggered upregulation of ATG4B at both mRNA and protein levels, and attenuated CRNDE-enhanced ATG4B mRNA stability in HCC cells. Luciferase reporter assay showed that overexpression of CRNDE increased the luciferase activity of pmir-ATG4B (Figure 5D), while silence of CRNDE decreased the luciferase activity of pmir-ATG4B (Figure 5E). Additionally, miR-543 markedly impaired the CRNDE-induced luciferase activity of pmir-ATG4B (Figure 5F). Moreover, compared to pcDNA-CRNDE, pcDNA-CRNDE-mut (in which the binding site of miR-543 was mutated) had no significant effect on miR-543 level (Figure 5G), but resulted in an attenuated induction of ATG4B and LC3-II, while caused a weakened reduction of SQSTM1/p62 (Figures 5H,I). These data indicate that CRNDE sequestrates miR-543, leading to the upregulation of ATG4B and enhancement of autophagy.

### CRNDE/ATG4B/Autophagy Pathway Alleviates the Sensitivity of Sorafenib in HCC Cells

**FIGURE 6:**
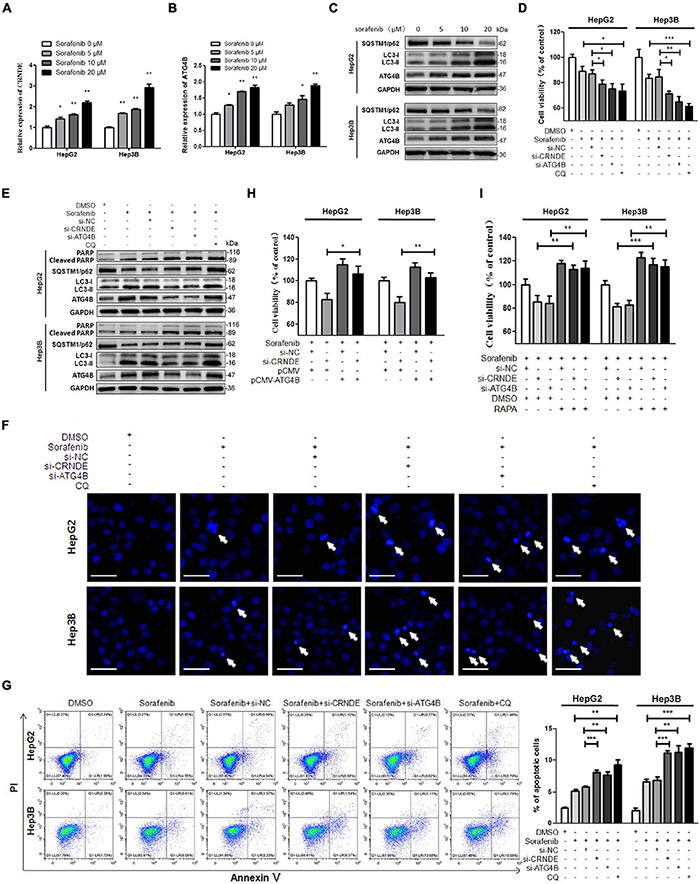
CRNDE/ATG4B/autophagy axis attenuates the sensitivity of sorafenib in HCC cells. **(A–C)** HepG2 and Hep3B cells were treated with various concentrations of sorafenib for 24 h. Then the levels of CRNDE and ATG4B mRNA were determined by qPCR **(A,B)**, and the levels of SQSTM1/p62, LC3, and ATG4B protein were measured by Western blot **(C)**. **(D,E)** HepG2 and Hep3B cells were transfected with si-CRNDE, si-ATG4B (or si-NC), or treated with 20 μM chloroquine (CQ) (or vehicle control DMSO) for 12 h, and then treated with 10 μM sorafenib for 24 h. Subsequently, the cell viability was analyzed by CCK-8 assay **(D)**, and the levels of cleaved PARP, SQSTM1/p62, LC3, and ATG4B were tested by Western blot **(E)**. **(F)** After treatment as in panels **(D,E)**, the cells were stained with Hoechst 33258, and then observed under a fluorescence microscope (scale bar: 20 μm; white arrows indicate the apoptotic cells). **(G)** After treatment as in panels **(D,E)**, the cells were stained with annexin V-FITC/PI, and then the cell apoptosis was assayed by flow cytometry. **(H)** After co-transfected with si-CRNDE (or si-NC) and pCMV-ATG4B (or pCMV) for 12 h, HepG2 and Hep3B cells were treated with 10 μM sorafenib for 24 h, and then the cell viability was detected by CCK-8 assay. **(I)** HepG2 and Hep3B cells were transfected with si-CRNDE, si-ATG4B (or si-NC) for 12 h, followed by the co-treatment with 0.1 μM rapamycin (RAPA) and 10 μM sorafenib (or vehicle control) for 24 h. Subsequently, the cell viability was examined by CCK-8 assay. si-CRNDE: the siRNA for CRNDE; si-ATG4B, si-NC, pCMV-ATG4B and pCMV were the same as the description in Figure 2; ns, no significance; *P < 0.05; **P < 0.01; ***P < 0.001.

As shown in Supplementary Figure 8, overexpression of CRNDE promoted the viability of HCC cells while silence of CRNDE suppressed the cell viability, which was consistent with the previous reports (Chen et al., 2016; Wang et al., 2018; Li et al., 2020). Besides, inhibition of autophagy with CQ significantly alleviated the CRNDE-promoted cell migration (Supplementary Figure 9). Since protective autophagy tends to weaken the anti-tumor effect of therapeutic reagents, we investigated whether the CRNDE-triggered autophagy influenced the response of HCC cells to sorafenib. Interestingly, treatment with sorafenib dramatically upregulated CRNDE and ATG4B, elevated LC3-II while reduced SQSTM1/p62 in HCC cells (Figures 6A–C). Additionally, knockdown of CRNDE or ATG4B, or inhibition of autophagy with CQ strengthened the suppression effect of sorafenib on cell survival (Figure 6D), and increased sorafenib-induced PARP cleavage (Figure 6E), apoptotic bodies (Figure 6F) and apoptotic cells (Figure 6G). Furthermore, overexpression of ATG4B markedly attenuated the reduction of cell survival caused by the co-treatment with si-CRNDE and sorafenib (Figure 6H). Meanwhile, the decrease of cell survival caused by the co-treatment with si-CRNDE (or si-ATG4B) and sorafenib was abolished by autophagy inducer rapamycin (RAPA; Figure 6I). Altogether, these results prove that sorafenib activates the CRNDE/ATG4B/autophagy pathway, which alleviates the sensitivity of sorafenib in HCC cells.

### Knockdown of CRNDE Enhances the Anti-HCC Effect of Sorafenib *in vivo*

**FIGURE 7:**
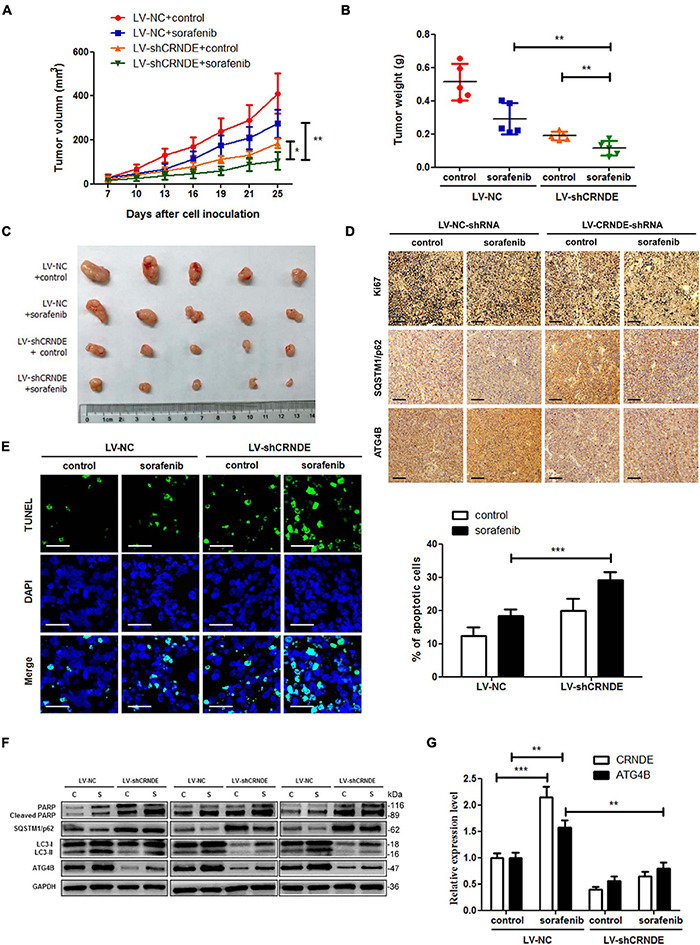
Knockdown of CRNDE sensitizes HCC cells to sorafenib *in vivo*. **(A–C)** The size, weight and photograph of xenograft tumors in the groups with or without stable knockdown of CRNDE (LV-shCRNDE or LV-NC) and administration of sorafenib (*n* = 5). **(D)** Immunohistochemical staining assay for Ki67, SQSTM1/p62 and ATG4B in the xenograft tumors of different groups as in panel **(A)** (scale bar: 100 μm). **(E)** TUNEL staining assay for apoptosis in the xenograft tumors of different groups as in panel **(A)** (scale bar: 20 μm). **(F)** Western blot analysis of cleaved PARP, SQSTM1/p62, LC3, and ATG4B in 3 xenograft samples of each group as in panel **(A)**. **(G)** qPCR analysis of CRNDE and ATG4B mRNA in the xenograft tumors of different groups as in panel **(A)**. C, control; S, sorafenib. *P < 0.05; **P < 0.01; ***P < 0.001.

To validate whether the above *in vitro* phenomenon is present *in vivo*, the cell models with (or without) stable knockdown of CRNDE were established (Supplementary Figure 10), and then the HCC xenograft experiments with the cell models in nude mice were performed. As shown in Figure 7, knockdown of CRNDE remarkably strengthened the sorafenib-mediated inhibition of xenograft tumor growth (Figures 7A–C) and decrease of the cell proliferation marker Ki67 (Figure 7D), while increased sorafenib-triggered cell apoptosis (Figure 7E) and PARP cleavage (Figure 7F). Additionally, sorafenib significantly elevated CRNDE (Figure 7G) and ATG4B (Figures 7D,F,G) in the xenograft tumors. Silence of CRNDE obviously attenuated the sorafenib-induced ATG4B (Figures 7D,F,G) and LC3-II (Figure 7F), whereas abolished the sorafenib-mediated reduction of SQSTM1/p62 (Figures 7D,F). These results demonstrate that knockdown of CRNDE strengthens the anti-HCC effect of sorafenib *in vivo*.

## Discussion

**FIGURE 8:**
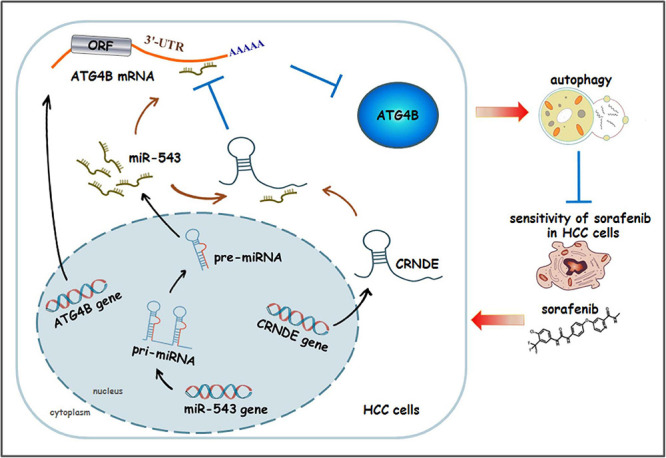
The schematic model illustrating how CRNDE induces autophagy and alleviates the anti-HCC effect of sorafenib. In this model, CRNDE sequestrates miR-543 and subsequently enhances ATG4B mRNA stability, leading to upregulaion of ATG4B and promotion of autophagy. Moreover, sorafenib activates CRNDE/ATG4B/autophagy pathway, which ultimately attenuates the sensitivity of sorafenib in HCC cells.

In the present study, we have revealed a novel signaling pathway CRNDE/ATG4B/autophagy, in which CRNDE upregulates ATG4B via sequestrating miR-543, leading to enhancement of ATG4B mRNA stability and autophagy in HCC cells. Additionally, sorafenib can activate this pathway, which eventually weakens the sensitivity of sorafenib in HCC cells. The schematic presentation summarizes how CRNDE attenuates the sensitivity of sorafenib in HCC cells via promoting ATG4B-mediated autophagy (Figure 8).

CRNDE displays a tissue-specific expression pattern, in which it has little or no expression in certain normal tissues such as liver. Several lines of evidence support the important role of CRNDE as a mediator of oncogenesis (Xie et al., 2018; Zhang et al., 2018). Here, we validated that CRNDE was aberrantly upregulated in HCC tissues and cell lines, which was consistent with prior studies (Chen et al., 2016; Wang et al., 2018; Li et al., 2020). A recent report has shown that silence of CRNDE promotes autophagy and cell viability in neurons exposed to hypoxic-ischemic (Fu et al., 2020). However, our study indicated that overexpression of CRNDE enhanced autophagy and cell viability in HCC cells. The discrepancy probably lies in the different pathological circumstances, which deserves indepth investigation to better understand the relationship between CRNDE and autophagy.

Numerous researches have highlighted regulation of ATG4B by enzymes, compounds, miRNAs, siRNAs, transcription factors and their binding proteins, and so on (Wu et al., 2016; Kjos et al., 2017; Zhang et al., 2017; Fu et al., 2019). Recently, two lncRNAs have been reported to promote autophagy and chemoresistance in colorectal cancer through miR-34a/ATG4B pathway (Li Y. et al., 2019; Liu et al., 2020). Nevertheless, it is still unclear whether CRNDE is associated with the regulation of ATG4B in HCC. In this study, we for the first time verified that CRNDE at least partially accounted for ATG4B upregulation and the subsequent autophagy induction in HCC cells, which disclosed a novel signaling pathway CRNDE/ATG4B/autophagy. In addition, as elevated CRNDE expression is typically found in a variety of malignancies, more studies are required to explore if the CRNDE-triggered upregulation of ATG4B exists functionally in other correlated neoplastic diseases.

RNA stability could be influenced by multiple factors such as RNA binding proteins, RNases and miRNAs (Wu et al., 2016). Recent studies have shown that CRNDE affects the expression and/or activity of some miRNAs as a sponge (Chen et al., 2016; Wang et al., 2018; Ji et al., 2019), while several miRNAs have impact on the stability of ATG4B mRNA in various cancer cells (Liao et al., 2016; Wu et al., 2016; Liu Y. et al., 2019). Here, miR-543 was screened out to be a candidate which might be involved in the CRNDE-mediated regulation of ATG4B, and subsequently this miRNA was proved to be necessary for CRNDE/ATG4B/autophagy signaling axis. Interestingly, compared to wild type CRNDE, mutation of CRNDE in the binding site of miR-543 led to an attenuated upregulation of ATG4B and LC3-II while a weakened reduction of SQSTM1/p62, suggesting that CRNDE might modulate ATG4B and the subsequent autophagy through other ways besides regulating miR-543. Additionally, the action mechanisms of miR-543 are also complicated. For instance, miR-543/TRPM7 axis participates in cervical cancer progression through PI3K/Akt and p38/MARK pathways (Liu X. et al., 2019). Another report has shown that miR-543/Angpt2 axis is involved in osteosarcoma metastasis and angiogenesis mediated by CTGF (Wang et al., 2017). Hence the other miR-543-associated signaling cascades as mentioned above might be affected by the CRNDE-caused change of miR-543, which needs further investigation to clarify the comprehensive intracellular signal networks relevant to CRNDE and miR-543.

Sorafenib, an oral multi-kinase inhibitor, has dual anti-tumor effects on both tumor cell proliferation and angiogenesis (Llovet et al., 2008). To date, sorafenib is regarded as the first approved and most widely-used systemic drug in the treatment of advanced HCC, but the therapeutic effect is less than satisfactory largely due to acquired drug resistance (Llovet et al., 2008; Sun et al., 2017). Mounting evidences have shown that protective autophagy weakens the lethality of sorafenib to HCC cells (Shi et al., 2011; Heqing et al., 2016). Mechanistically, sorafenib can induce autophagy in HCC cells through multiple ways, such as promoting IRE1 signals (Shi et al., 2011), activating Akt pathway (Zhai et al., 2014), suppressing mTORC1 (Shimizu et al., 2012), or inhibiting STAT3/Mcl-1/Beclin 1 axis (Tai et al., 2013), and so on. However, the detailed relationship between autophagy and sorafenib has not yet been clearly clarified. Although accumulating studies have reported the significant role of CRNDE in HCC, there is currently no evidence whether CRNDE affects the sensitivity of sorafenib in HCC cells. In the present study, we elucidated that sorafenib-activated CRNDE/ATG4B/autophagy pathway contributed to the reduced sensitivity of sorafenib in HCC cells, which is a novel role of CRNDE to induce sorafenib resistance. Nevertheless, it remains unknown how sorafenib elevates CRNDE level. Previous reports have shown that some factors such as RNA binding proteins, transcription factors, epigenetic modification, miRNAs, or specific signal pathways participate in the regulation of CRNDE (Wang et al., 2015; Huan et al., 2017; Jiang et al., 2017; Kiang et al., 2017; Ding et al., 2020). Sorafinib may upregulate CRNDE through one or several above factors, or other mechanism(s). Therefore, more studies are warranted regarding the detailed mechanisms by which sorafenib increases CRNDE in HCC cells.

In summary, our study presents the first evidence that CRNDE elevates ATG4B via sequestrating miR-543 and subsequently enhances ATG4B mRNA stability, which ultimately promotes autophagy in HCC cells. Moreover, sorafenib activates the CRNDE/ATG4B/autophagy pathway, and knockdown of CRNDE sensitizes HCC cells to sorafenib. These findings illustrate a key role of CRNDE in autophagy regulation and sorafenib resistance of HCC cells, suggesting that targeting the CRNDE/ATG4B/autophagy pathway may be a promising strategy to increase the sensitivity of sorafenib in HCC cells.

## Data Availability Statement

The original contributions presented in the study are included in the article/Supplementary Material, further inquiries can be directed to the corresponding authors.

## Ethics Statement

The studies involving human participants were reviewed and approved by The Ethics Committee of Army Medical University. The patients/participants provided their written informed consent to participate in this study. The animal study was reviewed and approved by Ethics Committee of Army Medical University.

## Author Contributions

LC and LS performed the experiments and drafted the manuscript. XD, TL, and XY participated in data curation and analysis. YuZ, HX, XS, and GH helped to investigation. WX and YaZ devoted to technical and material support. DT collected clinical samples. SY, YN, and XH revised and polished the manuscript. JL and FH contributed to study design and funding acquisition. All authors read and approved the final manuscript.

## Conflict of Interest

The authors declare that the research was conducted in the absence of any commercial or financial relationships that could be construed as a potential conflict of interest.

## Publisher’s Note

All claims expressed in this article are solely those of the authors and do not necessarily represent those of their affiliated organizations, or those of the publisher, the editors and the reviewers. Any product that may be evaluated in this article, or claim that may be made by its manufacturer, is not guaranteed or endorsed by the publisher.

## Footnotes

1
http://cancergenome.nih.gov/

2
http://starbase.sysu.edu.cn/

3
http://www.targetscan.org/vert_72/
