# Supplementary material for: LncRNA CRNDE Promotes ATG4B-Mediated Autophagy and Alleviates the Sensitivity of Sorafenib in Hepatocellular Carcinoma Cells
Source: Front Cell Dev Biol. 2021 Aug 2;9:687524. doi: 10.3389/fcell.2021.687524 (PMC8365422; doi:10.3389/fcell.2021.687524)
Supplement: Supplementary file 1 [file Data_Sheet_1.PDF]

Supplementary Material

Supplementary Tables

Supplementary Table 1. The primer sets used in qPCR.

	Targets	Forward primers (5′-3′)	Reverse primers (5′-3′)
1	CRNDE	AAATCAAAGTGCTCGAGTGGT	ACCTTCTTCTGCGTGACAAC
2	ATG4B	GGTGTGGACAGATGATCTTTGC	CCAACTCCCATTTGCGCTATC
3	GAPDH	ACGGATTTGGTCGTATTGGG	CGCTCCTGGAAGATGGTGAT
4	hsa-miR-543	HmiRQP0622, GeneCopoeia	
5	hsa-miR-455-3p	HmiRQP0515, GeneCopoeia	
6	hsa-miR-1197	HmiRQP0043, GeneCopoeia	
7	hsa-miR-126-5p	HmiRQP0098, GeneCopoeia	
8	hsa-miR-384	HmiRQP0482, GeneCopoeia	
9	hsa-miR-4436a	HmiRQP2061, GeneCopoeia	
10	hsa-miR-9-5p	HmiRQP0825, GeneCopoeia	
11	hsa-miR-665	HmiRQP0778, GeneCopoeia	
12	U6	HmiRQP9001, GeneCopoeia	

Supplementary Table 2. The sequences of siRNAs.

	siRNAs	Sequences (5′-3′)
1	si-ATG4B-1	GGUGUGGACAGAUGAUCUUUG
2	si-ATG4B-2	GCAUCUAGACUUUGGUUUA
3	si-ATG4B-3	CCAGGUCCUGAAGAAGCUU
4	si-CRNDE	GUGCUCGAGUGGUUUAAAU
5	si-NC	UUCUCCGAACGUGUCACGUTT

Supplementary Table 3. The sequences of miRNA mimics and inhibitors.

	RNA oligonucleotides	Sequences (5′-3′)
1	miR-543 mimics	AAACAUUCGCGGUGCACUUCUU
2	miR-NC	UCACAACCUCCUAGAAAGAGUAGA
3	miR-543 inhibitor	AAGAAGUGCACCGCGAAUGUUU
4	inhibitor-NC	UCUACUCUUUCUAGGAGGUUGUGA

Supplementary Figures

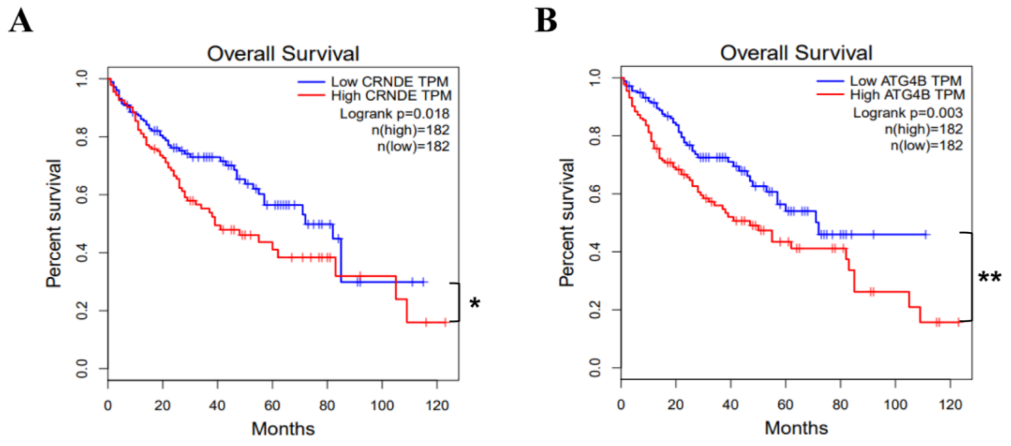

Supplementary Figure 1. CRNDE and ATG4B mRNA levels are related to a shorter overall survival of HCC patients. **(A,B)** The clinical data were from The Cancer Genome Atlas (TCGA) database. The overall survival of 364 HCC patients with high or low CRNDE (A) and ATG4B mRNA (B) levels were analyzed. $^{*}P<0.05$; $^{**}P<0.01$.

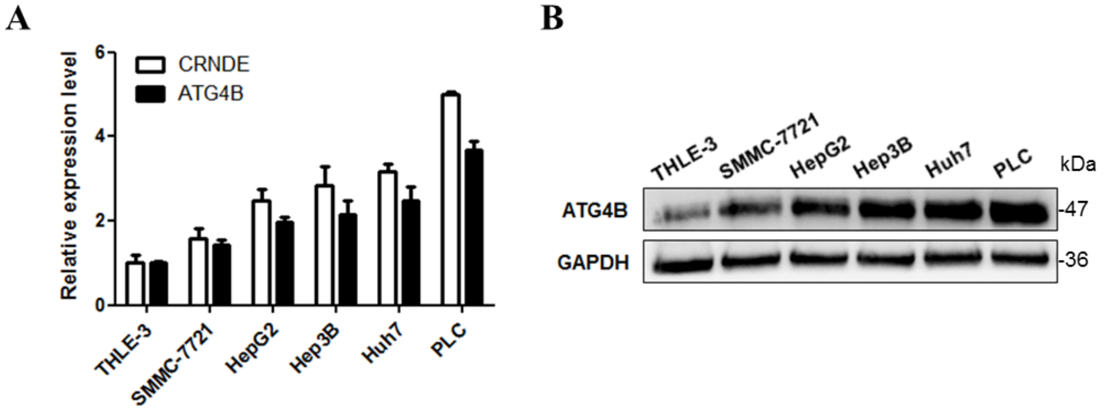

Supplementary Figure 2. The levels of CRNDE and ATG4B in HCC cell lines. **(A)** qPCR analysis of CRNDE and ATG4B mRNA levels in 5 HCC cell lines (SMMC-7721, HepG2, Hep3B, Huh7 and PLC) and relatively normal hepatic cell line THLE-3. **(B)** Western blot analysis of ATG4B protein level in the same cell lines as in A.

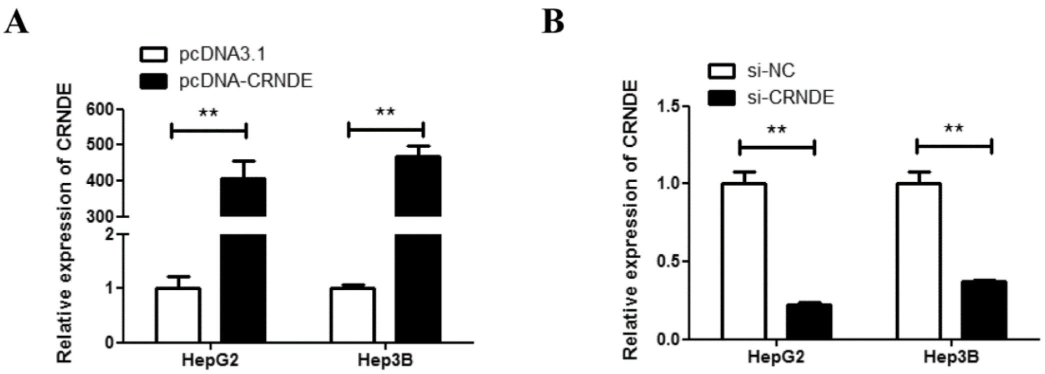

Supplementary Figure 3. Efficiency analysis of CRNDE expression vector and siRNA. **(A,B)** HepG2 and Hep3B cells were transfected with pcDNA-CRNDE (or pcDNA3.1) (A) or si-CRNDE (or si-NC) (B) for 24 h, then the level of CRNDE was detected by qPCR. pcDNA-CRNDE: CRNDE expression vector; pcDNA3.1: control vector pcDNA3.1(+); si-CRNDE: the siRNA for CRNDE; si-NC: control siRNA; $^{**}P$<0.01.

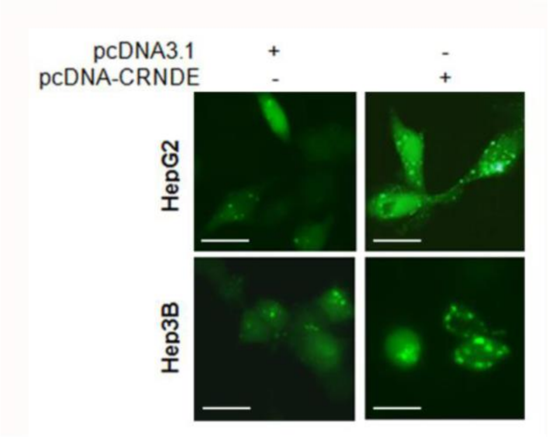

Supplementary Figure 4. CRNDE increases GFP-LC3 puncta in HCC cells. HepG2 and Hep3B cells were transfected with pcDNA-CRNDE (or pcDNA3.1) and GFP-LC3 vector for 24 h, then the green fluorescent GFP-LC3 puncta in the cells were observed under a fluorescence microscope (scale bar: 10 μm). pcDNA-CRNDE: CRNDE expression vector; pcDNA3.1: control vector pcDNA3.1(+).

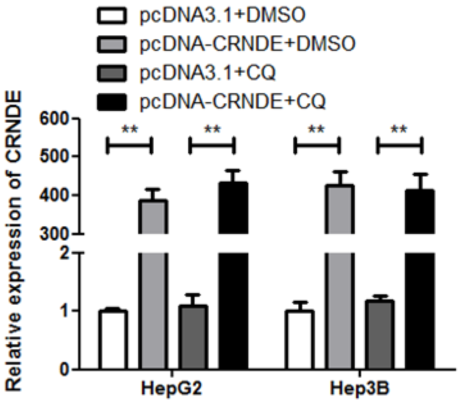

Supplementary Figure 5. Efficiency analysis of pcDNA-CRNDE in the presence of chloroquine (CQ). HepG2 and Hep3B cells were transfected with pcDNA-CRNDE (or pcDNA3.1) in the presence of 20 μM CQ (or vehicle control DMSO) for 24 h, and then the level of CRNDE was examined by qPCR. pcDNA-CRNDE: CRNDE expression vector; pcDNA3.1: control vector pcDNA3.1(+); $^{**}P$<0.01.

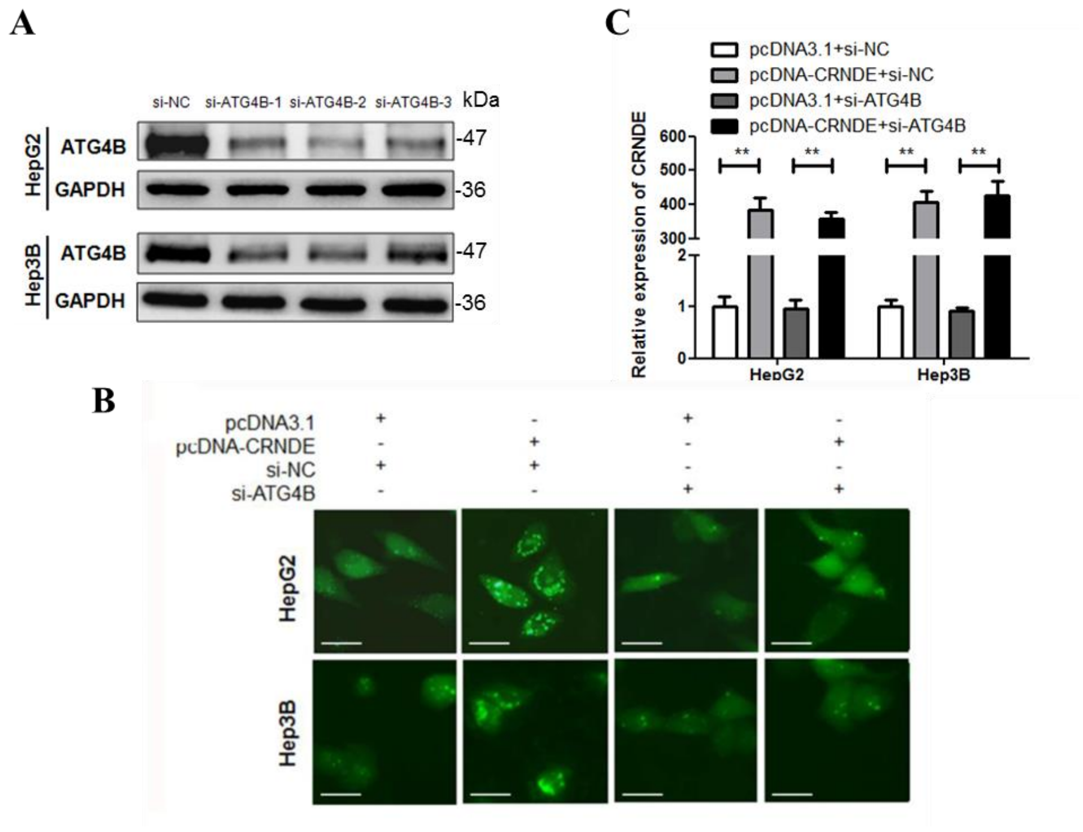

Supplementary Figure 6. CRNDE induces autophagy via upregulating ATG4B in HCC cells. **(A)** HepG2 and Hep3B cells were separately transfected with three different siRNAs of ATG4B (si-ATG4B-1, si-ATG4B-2, si-ATG4B-3 or si-NC) for 24 h, and then the level of ATG4B protein was determined by Western blot. **(B)** HepG2 and Hep3B cells were co-transfected with pcDNA-CRNDE (or pcDNA3.1) and si-ATG4B (or si-NC) in the presence of GFP-LC3 vector for 24 h, then the green fluorescent GFP-LC3 puncta in the cells were observed under a fluorescence microscope (scale bar: 10 μm). **(C)** HepG2 and Hep3B cells were co-transfected with pcDNA-CRNDE (or pcDNA3.1) and si-ATG4B (or si-NC) for 24 h, and then the level of CRNDE was measured by qPCR. pcDNA-CRNDE: CRNDE expression vector; pcDNA3.1: control vector pcDNA3.1(+); si-ATG4B: the siRNA for ATG4B; si-NC: control siRNA; $^{**}P$<0.01.

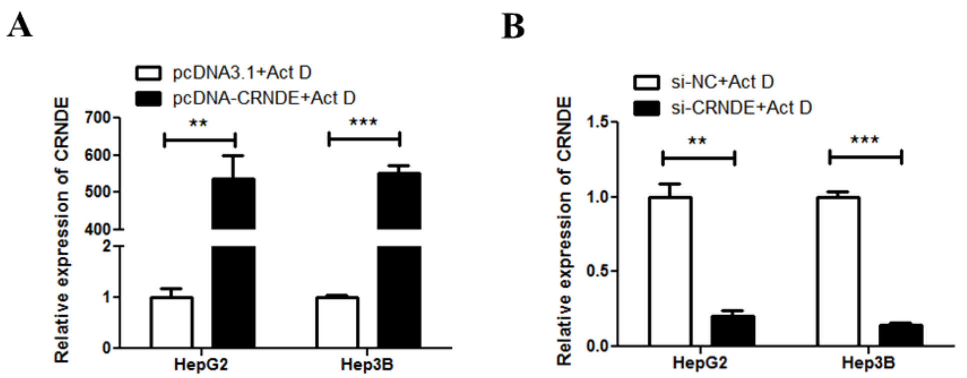

Supplementary Figure 7. Efficiency analysis of pcDNA-CRNDE and si-CRNDE in the presence of actinomycin D (Act D). **(A,B)** HepG2 and Hep3B cells were transfected with pcDNA-CRNDE (or pcDNA3.1) (A) or si-CRNDE (or si-NC) (B) for 18 h, followed by the treatment with 5 μg/mL Act D for 6 h. Then the level of CRNDE was detected by qPCR. pcDNA-CRNDE, pcDNA3.1, si-CRNDE and si-NC were the same as the description in Supplementary Figure 3; $^{**}P$<0.01; $^{***}P$<0.001.

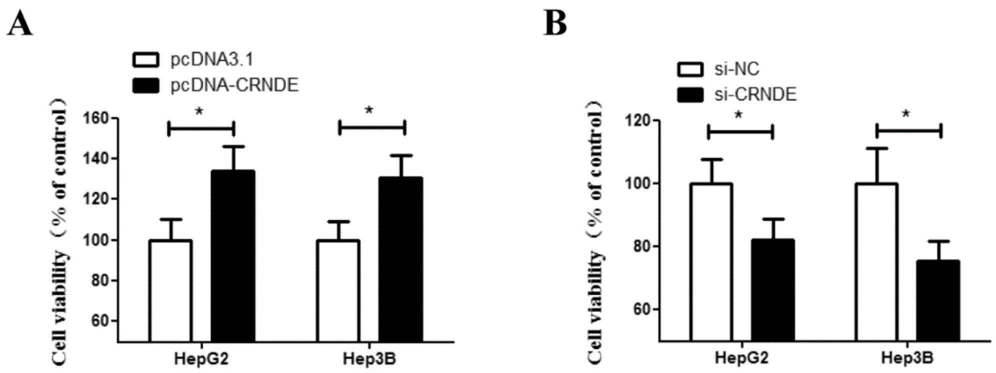

Supplementary Figure 8. CRNDE increases the viability of HCC cells. **(A,B)** HepG2 and Hep3B cells were transfected with pcDNA-CRNDE (or pcDNA3.1) (A) or si-CRNDE (or si-NC) (B) for 24 h, then the cell viability was measured by CCK-8 assay. pcDNA-CRNDE, pcDNA3.1, si-CRNDE and si-NC were the same as the description in Supplementary Figure 3; $^{*}P$<0.05.

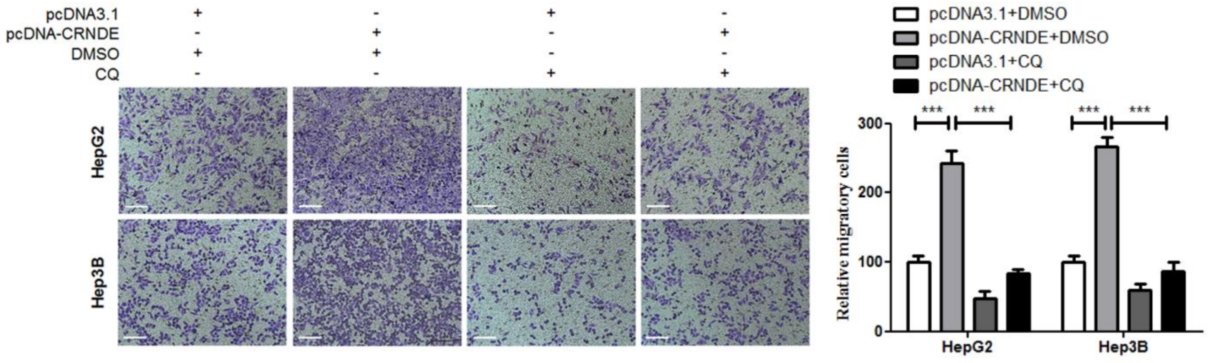

Supplementary Figure 9. Inhibition of autophagy alleviates CRNDE-promoted cell migration. HepG2 and Hep3B cells were transfected with pcDNA-CRNDE (or pcDNA3.1) in the presence of 20 μM chloroquine (CQ) (or vehicle control DMSO) for 24 h, then the cell migration was detected by transwell migration assay (scale bar: 100 μm). pcDNA-CRNDE: CRNDE expression vector; pcDNA3.1: control vector pcDNA3.1(+); $^{***}P<0.001$.

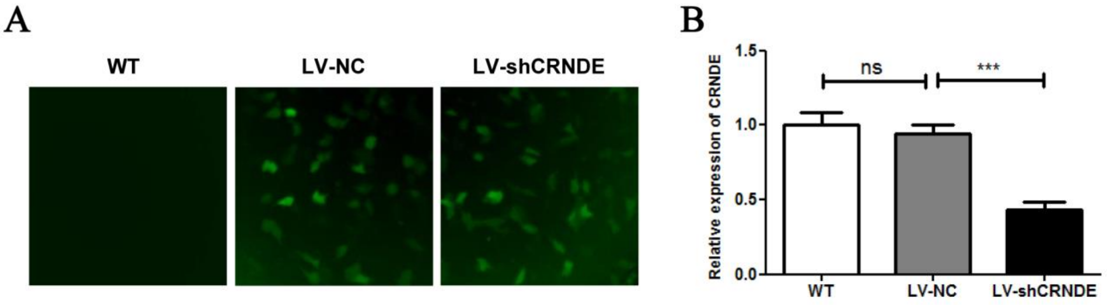

Supplementary Figure 10. Construction of HepG2 cell models with (or without) stable knockdown of CRNDE. **(A)** HepG2 cells were infected with the packaged lentiviral particles containing shRNA of CRNDE (LV-shCRNDE) or negative control (LV-NC), and then selected by puromycin. Subsequently, the screened cells (LV-shCRNDE and LV-NC) and the wild tpye (WT) HepG2 cells (as the blank control) were photographed under a fluorescence microscope (GFP as the detective marker). **(B)** qPCR analysis of CRNDE level in LV-shCRNDE, LV-NC and WT HepG2 cells. ns: no significance; $^{***}P<0.001$.
